# Differential Metabolomic Analysis of Liver Tissues from Rat Models of Parenteral Nutrition-Associated Liver Disease

**DOI:** 10.1155/2020/9156359

**Published:** 2020-03-21

**Authors:** Songlin Wan, Jianbo Yang, Gulsudum Mamtawla, Li Zhang, Xuejin Gao, Xinying Wang

**Affiliations:** Research Institute of General Surgery, Jinling Hospital, Medical School of Nanjing University, Nanjing 210002, China

## Abstract

Parenteral nutrition (PN) is a life-saving therapy for patients with intestinal failure, but parenteral nutrition-associated liver disease (PNALD) limits its long-term use. The present study is aimed at determining which pathways are altered most notably in a rat model of PNALD. We randomly assigned male Sprague-Dawley (SD) rats into two different groups, whereby they received either enteral nutrition (EN) or PN. Liver tissues were harvested from all rats 7 days later for metabolomic profiling. The composition of primary conjugated bile acids was altered, the synthesis of polyunsaturated fatty acids was reduced, the conversion of pyruvate to acetyl-CoA was blocked, and the synthesis of phosphatidylcholine was inhibited in rats with PNALD. Riboflavin, which is involved in the electron transfer process in the mitochondrial electron transport chain, was remarkably decreased in PNALD rats. A deficiency of polyunsaturated fatty acids, riboflavin, choline, and taurine might be involved in the progression of PNALD. The implications of these findings for the field of medicine are that supplementation with polyunsaturated fatty acids, riboflavin, choline, and taurine might have potential as therapeutic strategies for PNALD and also shed light on the mechanisms of PNALD.

## 1. Introduction

Parenteral nutrition (PN) refers to the intravenous delivery of nutrients such as amino acids, glucose, lipids, electrolytes, vitamins, and trace elements [1]. Parenteral nutrition is a life-saving therapy for patients who cannot tolerate enteral feeding [2]. However, parenteral nutrition-associated liver disease (PNALD; also known as intestinal failure-associated liver disease) that develops in many of such patients, especially infants and children, limits the long-term application of PN [3].

The main features of PNALD include cholestasis, hepatic steatosis, and fibrosis in the early stage, followed by disease progression, cirrhosis, and liver failure [4, 5]. Cholestasis, hepatic steatosis, and fibrosis associated with chronic PN progress to cirrhosis within 3–5 years in adults, but within 6–12 months in infants and young children with intestinal failure [6]. Therefore, PNALD is a major life threat to patients on long-term PN. El Kasmi et al. [6–8] reported that Kupffer cell activation by intestinal-derived lipopolysaccharide through Toll-like receptor 4 signaling, phytosterols from intravenous lipid emulsions, and macrophage-derived IL-1*β*/NF-*κ*B signaling promotes liver injury in PNALD. Furthermore, changes in metabolic processes such as lipogenesis, triglyceride secretion, and fatty acid oxidation contribute to the occurrence and progression of PNALD [9–11]. Despite considerable investigation, the cause(s) and mechanism(s) of the disease remain obscure.

The global and comprehensive analytical approach to identifying a set of metabolites in biochemical and biological samples, namely, metabolomics, provides the most downstream information about biochemical pathways by studying small organic molecules, and it has been applied to identify biomarkers and explain disease mechanisms.

Here, we aimed to determine which pathways are altered in the progression to PNALD and the molecular mechanisms involved in the development of PNALD, by comparing liver metabolic profiles between normal and PNALD models using coupled ultrahigh-performance liquid chromatography with quadrupole time-of-flight mass spectrometry (UHPLC-QTOF-MS) platform-based metabolomics.

## 2. Materials and Methods

### 2.1. Animal Model

The Institutional Animal Care and Use Committee of Jinling Hospital, Medical School of Nanjing University approved the animal experiments. Twelve 3-week-old male Sprague-Dawley rats (provided by the Animal Center of Jinling Hospital) were housed under specific pathogen-free conditions; they were acclimatized to this environment for seven days. Thereafter, the rats were randomized (*n* = 6 per group) to receive either enteral (EN) or parenteral (PN) nutrition. All rats were intraperitoneally anesthetized with avertin (150 mg/kg); then, central venous catheters (CVC) were inserted into the right external jugular veins. Normal saline was continuously administered through the catheters for 24 h during recovery from the surgical procedure. The EN group received standard laboratory chow and a continuous infusion of 0.9% saline, and the PN group received PN solution ([Supplementary-material supplementary-material-1]) prepared as described previously [12] with 0.9% saline infused at a rate of 265 mL/kg/24 h using a micropump (KD Scientific Inc., Holliston, MA, USA) through the CVC. The rats were sacrificed following anesthesia using avertin after seven days on each diet. Serum samples were collected and stored at -80°C. Liver samples were collected and immediately frozen in liquid nitrogen.

### 2.2. Metabolomic Analysis

Samples (25 mg) were added to EP tubes containing 1,000 *μ*L extract solution (acetonitrile : methanol : water, 2 : 2 : 1) and the internal standard, L-2-chlorophenylalanine (2 *μ*g/mL). The samples were Vortex-mixed, homogenized, sonicated in an ice-water bath, then incubated and centrifuged at 10000 rpm for 15 min at 4°C. Supernatants were decanted into fresh tubes, dried *in vacuo*. Then, the dried samples were reconstituted in 50% acetonitrile. The constitution was then centrifuged at 13000 rpm for 15 min at 4°C, and supernatant was transferred to a fresh glass vial for LC/MS analysis. The UHPLC separation proceeded using a 1290 Infinity series UHPLC System (Agilent Technologies Inc., Santa Clara, CA. USA), equipped with a UPLC 2.1 × 100 mm, 1.7 *μ*m, BEH Amide column (Waters Corp., Milford, MA, USA). The autosampler injection was divided into the positive and negative ion modes. We acquired MS/MS spectra on an information-dependent basis (IDA) during LC/MS experiments using a TripleTOF 6600 mass spectrometer (AB Sciex LLC). In this mode, Analyst TF 1.7 software (AB Sciex LLC) continuously evaluates the full scan-surveyed MS data as it collects and triggers the acquisition of MS/MS spectra depending on specified criteria.

### 2.3. Quantitative Detection of Liver Triglyceride and Malondialdehyde Levels

Triglyceride (TG) and malondialdehyde (MDA) concentrations were measured in liver tissues (~50 mg) using commercial assay kits (Jiancheng Bioengineering Institute, Nanjing, China) as described by the manufacturer.

### 2.4. Statistical Analysis

Raw MS data were processed using the R package XCMS (version 3.2). The process includes peak deconvolution, alignment, and integration. Minfrac and cutoff were set at 0.5 and 0.6, respectively. Metabolites were identified using the in-house MS2 database. Raw data were managed by single peak filtering, missing value recoding, and normalization with an internal standard. The internal structures of the data were determined using principal component analysis (PCA). Orthogonal variables among metabolites that were not associated with the categorical variables were filtered out, and those together with nonorthogonal variables were separately analyzed using orthogonal projections to latent structures-discriminant analysis (OPLS-DA) to increase the reliability of the metabolic information. All data are presented as mean ± standard deviation (SD). Statistical differences among metabolites were assessed using Student's *t* tests and the variable importance in projection (VIP) of the OPLS-DA mode. Values with *p* < 0.05 and VIP > 1 were regarded as being significant.

## 3. Results

**Figure 1:**
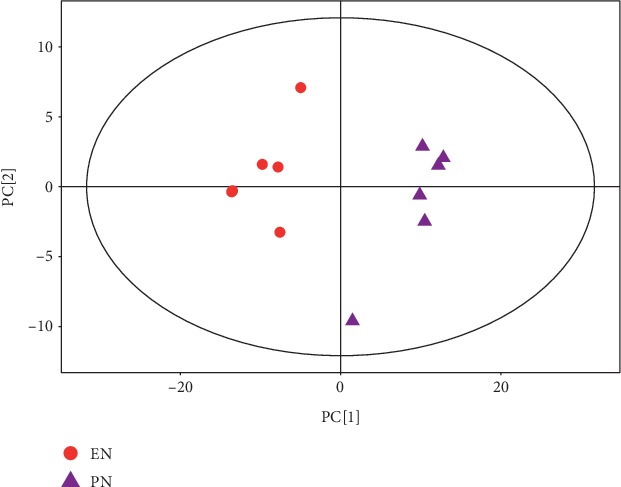
Scatter plots of PCA scores for EN vs. PN groups.

**Figure 2:**
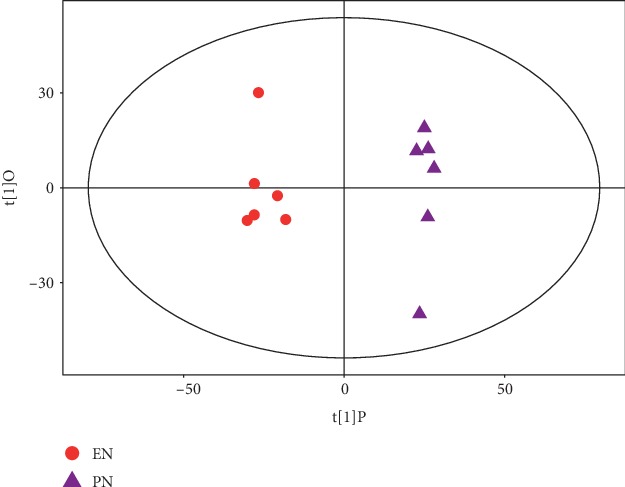
Scatter plot of OPLS-DA scores for EN vs. PN groups.

**Figure 3:**
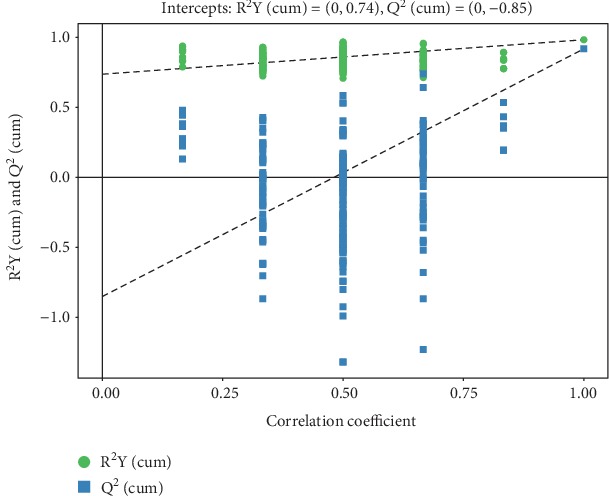
Permutation test of OPLS-DA model for EN vs. PN groups. The green dots and blue square points represent the R^2^Y values and *Q*^2^ values obtained from the displacement test, respectively. The two dashed lines represent the regression lines of R^2^Y and *Q*^2^.

The metabolic profiles in the livers from the PN to the EN groups significantly differed according to both PCA and OPLS-DA. PC1 explained 60% of the total variation (Figure 1). The OPLS-DA scores significantly differed between the groups (Figure 2), and the samples were all in the 95% confidence interval (CI; Hotelling's T-squared ellipse). The permutation results from the OPLS-DA model showed that the R^2^Y and *Q*^2^ values were 0.983 and 0.918, respectively (Figure 3).

We classified the metabolites into the functional categories of bile acids (BA), amino acids (AA), fatty acids (FA), and “other,” to explore potentially significant biochemical differences in PNALD progression in terms of metabolite class. Levels of triglycerides and MDA were significantly increased in the PN group (Figures [Supplementary-material supplementary-material-1] and [Supplementary-material supplementary-material-1]).

### 3.1. Bile Acid Metabolomics

Differentially expressed metabolite analysis revealed significant differences in the BA profiles between groups. Differentially expressed BA metabolites included three BA and three cholates (Table 1). The relative amounts of glycodeoxycholic, glycocholic and glycolithocholic acids, and glycochenodeoxycholate were significantly increased in the PN group. Conversely, taurochenodeoxycholate and taurocholate were significantly decreased in the PN group.

### 3.2. Amino Acid Metabolomics

**Figure 4:**
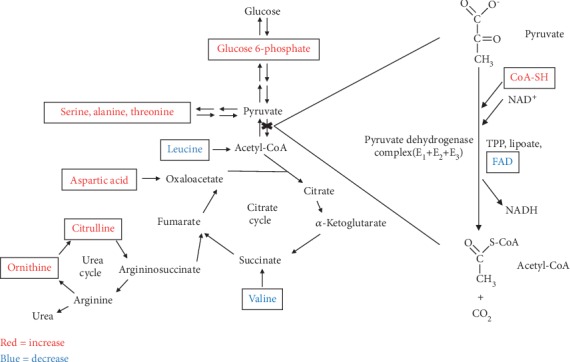
Amino acid-related metabolism pathway analysis. The red and blue colors depict amino acids showing a significant increase and decrease, respectively, in the PN group.

Nine AA metabolites significantly differed between the EN and PN groups (Table 2). The levels of serine, alanine, threonine, aspartic acid, ornithine, and citrulline increased, whereas those of leucine, valine, and taurine decreased in the PN group, compared with the EN group. The metabolic pathway analysis of differentially expressed AA metabolites showed important roles for the glycolytic pathway and the citric and urea cycles (Figure 4).

### 3.3. Fatty Acid Metabolomics

**Figure 5:**
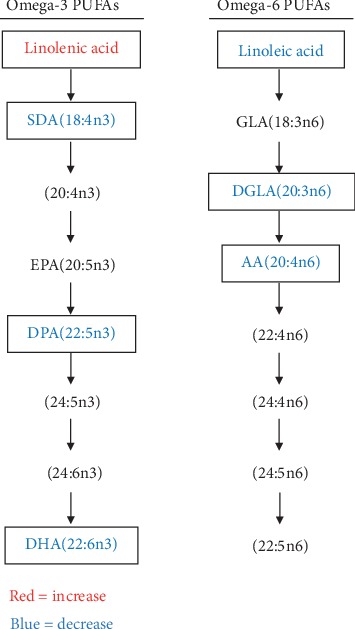
PUFA metabolism pathway analysis. The red and blue colors depict fatty acids showing a significant increase and decrease, respectively, in the PN group.

Twenty-six FA metabolites including 11 saturated and 15 unsaturated FA were differentially expressed between the two groups (Table 3). Compared with the EN group, the levels of 10 of the 15 unsaturated FA were significantly decreased, whereas five of them, including (Z)-6-octadecenoic acid, alpha-linolenic acid, oleic acid, 20-HETE, and 13(S)-HODE, were increased in the PN group. Metabolism pathway analysis revealed that the synthesis of polyunsaturated FA was changed in the PN group.  Figure 5 shows details of specific changes.

### 3.4. Other Metabolomics

**Figure 6:**
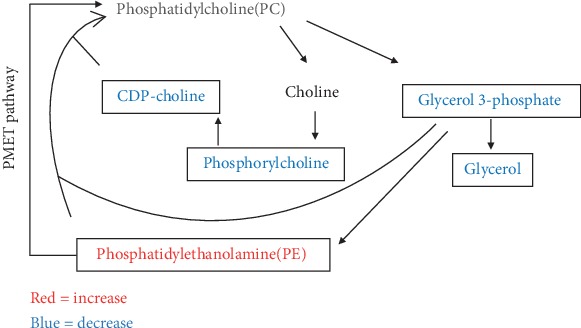
Glycerophospholipid metabolism pathway analysis. The red and blue colors depict compounds showing a significant increase and decrease, respectively, in the PN group.

Other metabolites of interest included acetylcarnitine, glycerol 3-phosphate, glycerol, CDP-choline, phosphatidylethanolamine, phosphorylcholine, flavin adenine dinucleotide (FAD), ubiquinone-10, and riboflavin (Table 4). Among them, acetylcarnitine, glycerol 3-phosphate, glycerol, CDP-choline, phosphorylcholine, FAD, and riboflavin were remarkably decreased, whereas phosphatidylethanolamine and ubiquinone-10 were increased in the PN group. The metabolic profiles of glycerophospholipid differed between the groups (Figure 6).

## 4. Discussion

The causes and mechanisms of PNALD, a critical and serious complication that limits long-term application of PN [3, 5], remain obscure despite a considerable amount of investigation. Metabolomics can identify biomarkers and reveal the mechanisms of disease and provide the most downstream information about biochemical pathways by studying small organic molecules. Changes in metabolic pathways that occur during PNALD progression can be ascertained by analyzing the metabolome. We found significant metabolic changes in bile, AA, and FA in the PN group. These changes involved BA profiles, glycolysis, the citric acid cycle, the urea cycle, the synthesis of polyunsaturated FA, and glycerophospholipid and riboflavin metabolism.

Cholestasis is one of the main characteristics of PNALD. Endogenous BA normally regulate cholesterol homeostasis, lipid solubilization, and metabolic signaling. Increased levels and an altered composition of BA in the liver can potentiate hepatotoxicity through activating inflammatory, oxidative stress, and necrotic cell death pathways [13, 14]. The accumulation of hydrophobic BA can induce mitochondrial damage, which leads to high levels of ROS production and oxidative stress [15]. Exposure to increased levels of hydrophobic BA also results in the direct activation of apoptosis and necrosis pathways [16, 17]. Almost 98% of hepatic BA are conjugated to either taurine or glycine in the liver, and thereafter, almost all of them present in a conjugated form [18]. We found that the levels of glycodeoxycholic acid, glycocholic acid, glycolithocholic acid, and glycochenodeoxycholate were significantly increased, whereas those of taurochenodeoxycholate and taurocholate were decreased in the PN group. All the BAs whose levels showed an increase and decrease were notably glycine- and taurine-conjugated, respectively. These findings were similar to those of a previous study of a rat model of NAFLD. Jia et al. found that total glyco-, rather than tauro-BA, was predominant in the livers of rats fed with a high fat-cholesterol (HFC) diet and positively correlated with macrovesicular steatosis scores [19]. In addition, glycochenodeoxycholic acid is considered the main toxic component of BA; it plays a critical role in the process of hepatocyte apoptosis in cholestatic liver injury [20]. We found 10-fold higher levels of GCDCA in the PN, than in the EN group. Unlike glyco-BAs, tauro-BAs are also considered to activate cell survival and antiapoptotic pathways to block their inherent toxicity *in vitro* and *in vivo* [21, 22]. The most likely mechanism of the changes in BA might have been associated with metabolic disorders of cholesterol and taurine, the latter of which was evident in the present study. These findings indicated that the changes in glycine and taurine conjugates, possibly resulting from cholesterol and taurine, play significant roles in the pathogenesis of PNALD.

The liver is the primary site of AA metabolism, which is thus in many liver diseases [23]. We found significant alterations in nine AA metabolites. Except for taurine and two types of branched-chain amino acids (leucine and valine) that were decreased in the PN group, all other AA species were increased. Analyses of the pathways of differentially expressed amino acid metabolites revealed important roles in the glycolytic pathway, as well as the citric acid and urea cycles. We inferred from the increased glucose 6-phosphate, serine, threonine, and alanine metabolites that the conversion of pyruvate to acetyl-CoA was blocked. The decreased leucine and valine and the increased aspartic acid, ornithine, and citrulline indicated inhibition of the citric acid cycle and subsequent activation of the urea cycle, which provides more support for a blocked process. Accumulated coenzyme A or CoA-SH, with reduced FAD and riboflavin (the precursor of FAD) that are important coenzymes in the pyruvate dehydrogenase complex also indicated blocked conversion of pyruvate to acetyl-CoA. However, whether Warburg-like effects are involved in the PNALD process requires further investigation.

The levels of not only taurine but also its precursor hypotaurine were reduced in the PN group. Taurine is involved in the formation of conjugated BA, and it contributes to numerous other biological functions through its antioxidative, anti-inflammatory, and membrane-stabilizing properties [24]. Taurine transporter knockout triggers chronic liver diseases, including hepatitis, liver fibrosis, and mitochondrial dysfunction [25]. Taurine supplementation can improve energy metabolism in liver-related diseases [24]. Therefore, a reduction in taurine and its metabolism might be involved in the pathogenesis of PNALD.

Polyunsaturated fatty acids (PUFA), mainly omega-6 and omega-3 PUFA, can influence the progression of fatty liver, hepatic steatosis, and hepatic microcirculation [26]. The present study found inhibited polyunsaturated FA metabolism in the PN group and significantly less arachidonic acid and DHA in the PN, than in the EN group. Arachidonic acid and DHA exert anti-inflammatory effects against liver injury or disease [27, 28]. The low levels of omega-3 PUFA in the PN group might also provide some evidence for treating of PNALD with fish oil [9, 29].

The metabolism of phosphatidylethanolamine (PE) and phosphatidylcholine (PC) is linked to the hepatocyte secretion of very low-density lipoproteins (VLDL) comprising triglycerides, cholesterol, and phospholipids. Impaired PC metabolism causes liver injury resulting from reduced VLDL synthesis and secretion [30, 31]. The synthesis of PC proceeds from choline via the dominant CDP-choline pathway and also from PE via the PE N-methyltransferase (PEMT) pathway [30]. Glycerol 3-phosphate, which can be hydrolyzed to generate glycerol, is a key precursor for PC biosynthesis. Glycerol 3-phosphate and PE participate in both pathways, whereas choline is involved only in the CDP-choline pathway. We found significantly more PE and significantly less glycerol 3-phosphate, CDP-choline, phosphorylcholine, and glycerol in the PN, than in the EN group. Our data and pathway analysis revealed that levels of PC might be lower in the PN, than in the EN group. The accumulation of PE and the deficiency of CDP-choline and phosphorylcholine show that the dominant synthesis pathway of PC is blocked.

FAD and ubiquinone-10 are involved in electron transfer within the mitochondrial electron transport chain. The water-soluble vitamin, riboflavin, is the precursor of flavin mononucleotide (FMN) and FAD. The decrease in riboflavin and FAD and the increase of ubiquinone-10 suggested a disorder of the electron transfer process, which can induce oxidative stress. Oxylipins originating from linoleic (e.g., 13(S)-HODE) and arachidonic (e.g., 20-HETE) acids were significantly elevated, suggesting lipid peroxidation in the PN group. However, this could also be a consequence of altered CYP450 activity [32]. Consequently, we further assessed the amount of MDA in liver tissue, which is one of the most important products of lipid peroxidation. As shown in [Supplementary-material supplementary-material-1], the amount of MDA was increased in liver tissues from the PN group. These results again suggest that oxidative stress is involved in PNALD.

## 5. Conclusions

We used ultrahigh-performance liquid chromatography coupled with quadrupole time-of-flight mass spectrometry (UHPLC-QTOF-MS) platform-based metabolomics to compare metabolomics between normal and PNALD model rats. The results showed that a deficiency of polyunsaturated FA, riboflavin, choline, and taurine might be involved in the progression of PNALD. Our findings also suggested that supplementation with polyunsaturated FA, riboflavin, choline, and taurine might have potential as therapeutic strategies for PNALD and also shed light on the mechanisms of PNALD.

## Figures and Tables

**Table 1:** Differentially expressed bile acid metabolites between the EN and PN groups.

Metabolites	EN	PN	*p* value
Glycodeoxycholic acid	0.22 ± 0.35	3.54 ± 2.67	0.028
Glycocholic acid	0.23 ± 0.31	3.52 ± 2.06	0.011
Glycolithocholic acid	0.0020 ± 0.0007	0.0073 ± 0.0048	0.044
Glycochenodeoxycholate	0.0032 ± 0.0043	0.033 ± 0.020	0.013
Taurochenodeoxycholate	0.25 ± 0.06	0.082 ± 0.087	0.003
Taurocholate	21.76 ± 8.53	4.44 ± 3.07	0.003

Bile acid metabolite values represent the mean relative amount to an internal standard ± the standard deviation.

**Table 2:** Differentially expressed amino acid metabolites between the EN and PN groups.

Metabolites	EN	PN	*p* value
L-Serine	0.23 ± 0.04	0.39 ± 0.05	0.000
L-Leucine	1.84 ± 0.16	1.57 ± 0.23	0.042
L-Alanine	0.58 ± 0.08	0.73 ± 0.05	0.003
L-Threonine	0.031 ± 0.008	0.067 ± 0.021	0.003
L-Valine	0.016 ± 0.007	0.0054 ± 0.0015	0.010
D-Aspartic acid	0.033 ± 0.004	0.047 ± 0.013	0.041
Ornithine	0.023 ± 0.006	0.032 ± 0.005	0.013
Citrulline	0.073 ± 0.014	0.099 ± 0.013	0.007
Taurine	2.93 ± 1.22	0.85 ± 0.38	0.007

Amino acid metabolite values represent the mean relative amount to an internal standard ± the standard deviation.

**Table 3:** Differentially expressed fatty acid metabolites between the EN and PN groups.

Metabolites	EN	PN	*p* value
Tridecanoic acid (tridecylic acid)	0.26 ± 0.04	0.16 ± 0.05	0.004
Tricosanoic acid	0.075 ± 0.055	0.22 ± 0.08	0.004
Tetracosanoic acid	0.33 ± 0.27	0.85 ± 0.36	0.017
Pentadecanoic acid	5.27 ± 1.61	2.11 ± 0.47	0.004
Palmitic acid	345.03 ± 88.89	156.27 ± 26.58	0.003
Myristic acid	0.14 ± 0.06	0.079 ± 0.030	0.044
Heptadecanoic acid	2.55 ± 0.57	1.12 ± 0.19	0.001
Dodecanoic acid	2.62 ± 0.27	1.72 ± 0.43	0.001
Arachidic acid	0.34 ± 0.14	0.53 ± 0.12	0.035
Behenic acid	0.044 ± 0.047	0.14 ± 0.08	0.027
Stearic acid	0.027 ± 0.020	0.14 ± 0.10	0.035
Linoleic acid	199.58 ± 54.27	114.23 ± 36.43	0.010
cis-9-Palmitoleic acid	23.25 ± 6.91	4.97 ± 1.19	0.001
Arachidonic acid (peroxide free)	85.28 ± 19.76	37.86 ± 14.85	0.001
Adrenic acid	19.15 ± 5.48	3.80 ± 0.95	0.001
7Z,10Z,13Z,16Z,19Z-Docosapentaenoic acid	27.01 ± 6.74	5.81 ± 1.24	0.000
2E-Eicosenoic acid	2.90 ± 1.02	1.52 ± 0.22	0.020
11(Z),14(Z)-Eicosadienoic acid	7.78 ± 2.37	2.64 ± 0.39	0.003
(4Z,7Z,10Z,13Z,16Z,19Z)-4,7,10,13,16,19-Docosahexaenoic acid	75.87 ± 13.18	22.98 ± 8.71	0.000
(Z)-6-Octadecenoic acid	0.0038 ± 0.0013	0.052 ± 0.019	0.001
Alpha-linolenic acid	0.041 ± 0.007	1.23 ± 0.28	0.000
Dihomo-gamma-linolenic acid	10.64 ± 2.90	3.97 ± 1.13	0.000
Oleic acid	0.0059 ± 0.0019	0.18 ± 0.09	0.004
Stearidonic acid	0.99 ± 0.26	0.48 ± 0.13	0.002
20-HETE	0.0055 ± 0.0035	0.10 ± 0.02	0.000
13(S)-HODE	0.058 ± 0.023	5.44 ± 1.95	0.001

Fatty acid metabolite values represent the mean amount relative to an internal standard ± the standard deviation.

**Table 4:** Other metabolomic changes in the EN and PN groups.

Metabolites	EN	PN	*p* value
Acetylcarnitine	0.84 ± 0.37	0.21 ± 0.10	0.008
CoA-SH	0.079 ± 0.071	0.51 ± 0.17	0.043
Glycerol 3-phosphate	8.87 ± 0.92	7.21 ± 1.54	0.047
Glycerol	0.011 ± 0.001	0.0072 ± 0.0007	0.002
Cytidine 5′-diphosphocholine (CDP-choline)	0.71 ± 0.11	0.49 ± 0.12	0.008
Phosphatidylethanolamine	0.020 ± 0.012	0.051 ± 0.020	0.008
Phosphorylcholine	0.26 ± 0.05	0.19 ± 0.04	0.028
Flavin adenine dinucleotide (FAD)	0.043 ± 0.004	0.035 ± 0.006	0.026
Ubiquinone-10 (coenzyme Q10)	0.024 ± 0.010	0.044 ± 0.013	0.014
Riboflavin	4.86 ± 1.20	0.91 ± 0.17	0.000

Metabolite values represent the mean relative amount to an internal standard ± the standard deviation.

## Data Availability

The data used to support the findings of this study are included within the article.

## Supplementary Materials

Supplementary Materials Table S1 provided the precise information of the composition of PN solution. PN solution contains all the nutrients needed for life activities such as amino acids, glucose, lipids, electrolytes, vitamins, and trace elements. The calorie percentages of glucose, lipid, and amino acid in PN solution were 59.1, 14.1, and 26.7, respectively. Figure S1 shows the triglyceride levels in liver tissue between the two groups. Compared to the EN group, the level of triglycerides was significantly increased in the PN group (15.45 ± 1.46 nmol/mg liver vs. 52.92 ± 5.10 nmol/mg liver, *p* < 0.05). Figure S2 shows the MDA levels in liver tissue between the two groups. Compared to the EN group, the level of MDA was significantly increased in the PN group (13.34 ± 1.64 nmol/g liver vs. 32.97 ± 2.49 nmol/g liver, *p* < 0.05).
