# Supplementary material for: Differential Metabolomic Analysis of Liver Tissues from Rat Models of Parenteral Nutrition-Associated Liver Disease
Source: Biomed Res Int. 2020 Mar 21;2020:9156359. doi: 10.1155/2020/9156359 (PMC7115143; doi:10.1155/2020/9156359)

Table S1: Nutrient formula for Parenteral Nutrition Solution.

| **Component** | **Amount, per liter** |
| --- | --- |
| Dextrose | 187.0 g |
| Amino acids | 44.7 g |
| Lipids | 37.6 g |
| Sodium chloride | 1.6 g |
| Sodium phosphate | 5.5 g |
| Potassium chloride | 1.0 g |
| Calcium gluconate | 2.3 g |
| Potassium acetate | 3.3 g |
| Magnesium sulfate | 0.8 g |
| Manganese | 0.8 mg |
| Zinc | 2.0 mg |
| Vitamin C | 200 mg |
| Thiamine | 6 mg |
| Riboflavin | 3.6 mg |
| Pyridoxine HCl | 6 mg |
| Niacinamide | 40 mg |
| Folic acid | 600 μg |
| Biotin | 60 μg |
| Cyanocobalamin | 5 μg |
| Vitamin K1 | 150 μg |
| Copper | 0.5 μg |
| Vitamin A | 3300 IU |
| Vitamin D3 | 200 IU |
| Vitamin E | 10 IU |

Figure S1: The triglyceride levels in liver tissue.


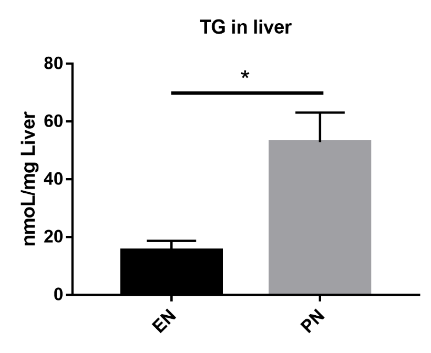


Figure S2: The malondialdehyde levels in liver tissue.


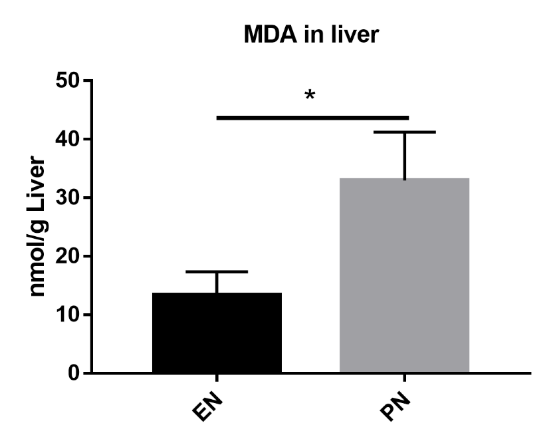

Supplement: Supplementary Materials — Table S1 provided the precise information of the composition of PN solution. PN solution contains all the nutrients needed for life activities such as amino acids, glucose, lipids, electrolytes, vitamins, and trace elements. The calorie percentages of glucose, lipid, and amino acid in PN solution were 59.1, 14.1, and 26.7, respectively. Figure S1 shows the triglyceride levels in liver tissue between the two groups. Compared to the EN group, the level of triglycerides was significantly increased in the PN group (15.45 ± 1.46 nmol/mg liver vs. 52.92 ± 5.10 nmol/mg liver, p < 0.05). Figure S2 shows the MDA levels in liver tissue between the two groups. Compared to the EN group, the level of MDA was significantly increased in the PN group (13.34 ± 1.64 nmol/g liver vs. 32.97 ± 2.49 nmol/g liver, p < 0.05). [file 9156359.f1.docx]
